# 1201. Putting a CAP on Discharge Antimicrobial Therapy: Evaluation of a Systematic Transitions of Care Process for Patients with Community Acquired Pneumonia (CAP) and Chronic Obstructive Pulmonary Disease (COPD)

**DOI:** 10.1093/ofid/ofad500.1041

**Published:** 2023-11-27

**Authors:** Ana Christine Belza, Jessica Efta, Nancy MacDonald, Rachel M Kenney, Nisha Patel

**Affiliations:** Henry Ford Health - Detroit, Fraser, Michigan; Henry Ford Health, Brownstown, Michigan; Henry Ford Hospital, Washington Twp, Michigan; Henry Ford Hospital, Washington Twp, Michigan; Henry Ford Hospital, Washington Twp, Michigan

## Abstract

**Background:**

Prescribing excess antibiotic duration at hospital discharge is common. A collaborative, pharmacist-led Antimicrobial Stewardship Transition of Care (ASP TOC) intervention implemented in our hospital was associated with improved discharge prescribing and reduced patient harm. However, the sustainability of this pharmacy service was challenging. To improve sustainability, the electronic scoring system (ESS) in the electronic medical record, used by inpatient pharmacists to prioritize patient care and identify interventions, had implemented an ASP TOC decision support in 2023. The purpose of this study was to evaluate the implementation of ASP TOC in the ESS.

**Methods:**

This IRB-approved retrospective quasi-experiment included patients discharged on oral antibiotics for community-acquired pneumonia or chronic obstructive pulmonary disease exacerbation (LRTI) from 11/2021 to 2/2022 (pre-intervention) or 11/2022 to 2/2023 (post-intervention). The primary endpoint was optimized discharge antimicrobial regimen, defined as guideline concordant selection, dose, and duration. 194 patients were required to achieve 80% power and detect 20% reduction of non-optimized therapy. Multivariable logistic regression was used to identify factors associated with optimized regimens.

**Results:**

200 patients were included. Similar baseline demographics were found in both the pre-intervention group and post-intervention group (Table 1). Optimized discharge regimens improved from 69% in the pre-intervention group to 82% in the post-intervention group (p = 0.033). ASP TOC interventions by the pharmacist increased from 4% to 25% in the post-intervention group (p < 0.001). After adjustment for the type of LRTI, ASP TOC intervention was independently associated with optimized discharge regimens (aOR 6.57; 95% CI 1.51-28.63).

**Table 1.**
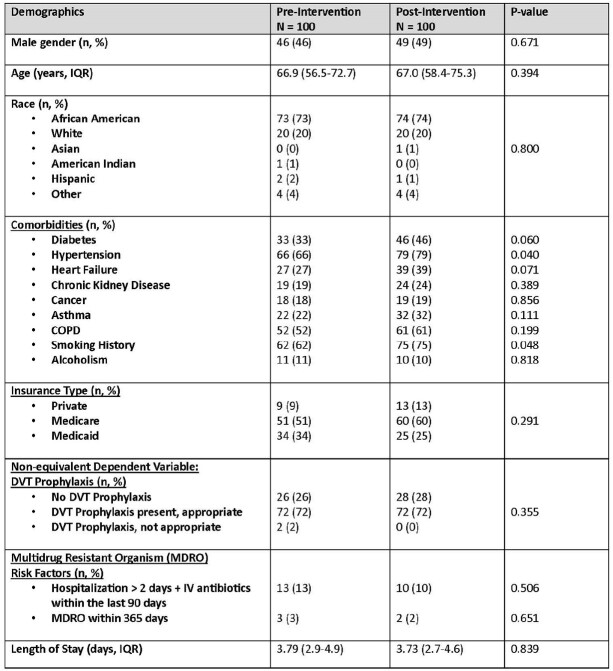
Baseline Demographics

**Conclusion:**

After launch of the ASP TOC decision support, there was an increase in optimized discharge regimens and ASP TOC interventions completed. Pharmacist use of ASP TOC decision support through an ESS can aid in improving discharge prescribing by the primary medical team, leading to improved outcomes.

**Disclosures:**

**All Authors**: No reported disclosures

